# Clinicopathological and mutational analyses of colorectal cancer with mutations in the *POLE* gene

**DOI:** 10.1002/cam4.2344

**Published:** 2019-06-25

**Authors:** Hitoshi Hino, Akio Shiomi, Masatoshi Kusuhara, Hiroyasu Kagawa, Yushi Yamakawa, Keiichi Hatakeyama, Takanori Kawabata, Takuma Oishi, Kenichi Urakami, Takeshi Nagashima, Yusuke Kinugasa, Ken Yamaguchi

**Affiliations:** ^1^ Division of Colon and Rectal Surgery Shizuoka Cancer Center Hospital Shizuoka Japan; ^2^ Regional Resources Division Shizuoka Cancer Center Research Institute Shizuoka Japan; ^3^ Medical Genetics Division Shizuoka Cancer Center Research Institute Shizuoka Japan; ^4^ Clinical Research Promotion Unit, Clinical Research Center Shizuoka Cancer Center Shizuoka Japan; ^5^ Division of Pathology Shizuoka Cancer Center Hospital Shizuoka Japan; ^6^ Cancer Diagnostics Research Division Shizuoka Cancer Center Research Institute Shizuoka Japan; ^7^ SRL Inc Tokyo Japan; ^8^ Department of Gastrointestinal Surgery Tokyo Medical and Dental University Tokyo Japan; ^9^ Shizuoka Cancer Center Hospital and Research Institute Shizuoka Japan

**Keywords:** colorectal cancer, DNA polymerase epsilon gene, hypermutation, ultramutation

## Abstract

Here, we investigated the clinicopathological and mutation profiles of colorectal cancer (CRC) with *POLE* mutations. Whole‐exome sequencing was performed in 910 surgically resected primary CRCs. Tumors exceeding 500 counts of nonsynonymous single nucleotide variants (SNVs) were classified as hypermutators, whereas the remaining were classified as nonhypermutators. The hypermutators were subdivided into 2 groups. CRCs harboring more than 20% C‐to‐A and less than 3% C‐to‐G transversions were classified as POLE category tumors, whereas the remaining were classified as common‐hypermutators. Gene expression profiling (GEP) analysis was performed in 892 (98.0%) tumors. Fifty‐seven (6.3%) and 10 (1.1%) tumors were classified common‐hypermutators and POLE category tumors, respectively. POLE category tumors harbored a significantly higher SNV count than common‐hypermutators, and all POLE category tumors were associated with exonuclease domain mutations, such as P286R, F367C, V411L, and S297Y, in the *POLE* gene. Patients with POLE category tumors were significantly younger than those with nonhypermutators and common‐hypermutators. All *POLE* mutations in the early‐onset (age of onset ≤50 years old) POLE category (7 tumors) were P286R mutations. GEP analysis revealed that *PD‐L1* and *PD‐1* gene expression levels were significantly increased in both common‐hypermutators and POLE category tumors compared with those in nonhypermutators.* CD8A* expression was significantly upregulated in POLE category tumors compared with that in nonhypermutators. Thus, we concluded that CRCs with POLE proofreading deficiency had characteristics distinct from those of other CRCs. Analysis of POLE proofreading deficiency may be clinically significant for personalized management of CRCs.

## 1 INTRODUCTION

Colorectal cancers (CRCs) can be split into 2 major groups according to tumor mutation rates in The Cancer Genome Atlas Network.^1^ Eighty‐four percent of CRCs have tumor mutation rates of less than 8.24/megabase (Mb), and the remaining 16% have tumor mutation rates of greater than 12/Mb; these tumors are classified as nonhypermutated and hypermutated cancers, respectively. Furthermore, hypermutated tumors can be further subdivided into 2 subsets; a small subset, (3% of CRCs) has an extremely high tumor mutation rate and is called ultramutated cancers.^2^

Proofreading by DNA polymerases and the function of DNA mismatch repair (MMR) facilitate high‐fidelity DNA replication in human cells. During the proof reading process, DNA polymerase epsilon (POLE) and DNA polymerase delta (POLD1) have central roles in replicating the leading and lagging DNA strands, respectively.^3^, ^4^, ^5^ Mutations in the exonuclease domain in *POLE* lead to impaired proofreading function, resulting in massively increased tumor mutation burden (TMB).^1^, ^6^, ^7^

Ultramutated CRCs have exonuclease domain mutations in *POLE*,^1^, ^2^, ^8^ and tumors with these mutations harbor a characteristic nucleotide substitution spectrum with a high frequency of C‐to‐A transversions.^1^, ^7^ Previous studies have shown that patients with endometrial cancer (EC) and glioblastoma harboring pathogenic exonuclease domain mutations in the *POLE* gene exhibit better prognosis,^9^, ^10^ suggesting that these mutations may be promising prognostic biomarkers. In addition, patients with EC harboring these mutations may be indicated for treatment with immune checkpoint inhibitors.^11^, ^12^ However, the clinical significance of *POLE* mutations in CRCs is less clear.

Here, we performed comprehensive genetic profiling of primary CRCs using whole‐exome sequencing (WES) and gene expression profiling (GEP) analysis in a large Japanese population. The association between *POLE* mutations, particularly exonuclease domain mutations, and the clinicopathological factors and gene expression profiles of primary CRCs were investigated to evaluate the clinical significance of these mutations.

## 2 MATERIALS AND METHODS

### 2.1 Ethical statement

To investigate the biological characteristics of cancer and diathesis of each patient with cancer, Shizuoka Cancer Center started Project HOPE (High‐tech Omics‐based Patient Evaluation) in 2014.^13^ In this project, multiomics‐based analyses, which integrated genomics, transcriptomics, proteomics, and metabolomics, were performed for various types of cancer with the goal of advancing cancer medicine. Project HOPE was designed according to the “Ethical Guidelines for Human Genome and Genetic Analysis Research” revised in 2013.^13^ Written consent was always obtained from patients participating in Project HOPE. The present study used the data from Project HOPE, and was approved by the Institutional Review Board of Shizuoka Cancer Center (approval no.29‐J74‐29‐2‐3).

### 2.2 Patient selection and study design

**Figure 1:**
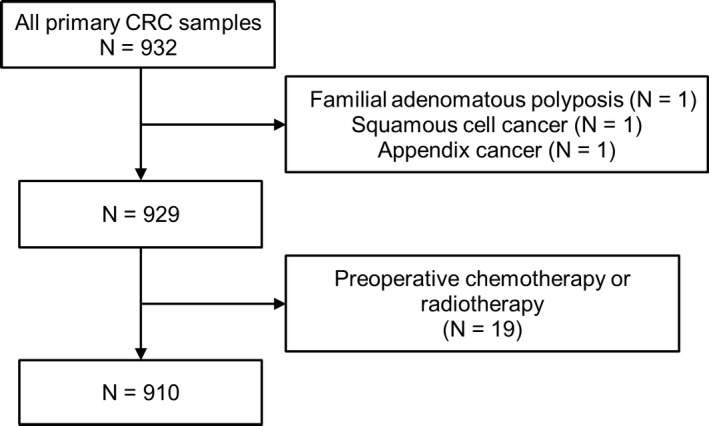
Flow diagram of tumor tissue selection. CRC: colorectal cancer

The candidates for Project HOPE were patients who underwent surgery to remove cancers at Shizuoka Cancer Center Hospital and who could supply fresh cancer tissues with sufficient quantity. Patients with tumors for whom the pathological diagnosis may be affected by the removal of a sufficient quantity of cancer tissue were excluded from this study. From January 2014 to February 2017, 932 primary CRCs was analyzed in Project HOPE. Patients who had familial adenomatous polyposis (n = 1), squamous cell cancer (n = 1), or appendix cancer (n = 1) or who underwent preoperative chemotherapy or radiotherapy (n = 19) were then excluded. Finally, 910 tumor samples were eligible for inclusion the present study (Figure 1). All tumor tissues were pathologically diagnosed as adenocarcinoma. Clinicopathological and genomic factors of these CRCs were analyzed retrospectively. Some of the eligible tumor samples in this study were also included in a previous report of *POLE* mutations, which described many types of tumors and focused on mutation‐driven tumorigenesis.^14^ In this study, the associations between *POLE* mutations and clinicopathological factors and between gene expression profiles and tumor immune responses were newly investigated in a larger number of CRCs.

### 2.3 Clinical samples

Approximately 0.1 g or more of cancer tissues was necessary for subsequent analysis. Tumor tissue samples were dissected from fresh surgical specimens. The surrounding normal tissue was also obtained whenever possible. In addition, peripheral blood was collected as a control for WES. For DNA analysis, dissected tissue and blood samples were immediately frozen in liquid nitrogen before DNA extraction. DNA was extracted from tissue samples using a QIAamp DNA Blood MINI Kit (Qiagen, Venlo, the Netherlands). DNA was quantified using a NanoDrop and Qubit 2.0 Fluorometer (Thermo Fisher Scientific, Waltham, MA). For RNA analysis, tissue samples were submerged in RNAlater solution (Thermo Fisher Scientific), minced, and stored at 4°C overnight before RNA extraction.

### 2.4 WES analysis of CRC tissues using next‐generation sequencing

WES analysis was performed as previously described.^14^, ^15^, ^16^ Briefly, DNA was subjected to WES on an Ion Proton System (Thermo Fisher Scientific). Torrent Suite software (ver. 4.4; Thermo Fisher Scientific) was used to convert binary raw data into sequence reads that were mapped to the reference human genome (UCSC, hg19). At this step, sequence data derived from tumor and blood samples were analyzed individually. The mapping results were stored as BAM files. Two BAM files uploaded to the Ion Reporter system were analyzed simultaneously. For this analysis, AmpliSeq exome tumor‐normal pair workflow (ver. 4.4, Thermo Fisher Scientific) with a Custom Hotspot file was used, and this Custom Hotspot file specifies the somatic and pathogenic mutations registered in COSMIC and ClinVar. The sequence data derived from blood samples were used as matched controls, and mutations identified in tumor samples but not detected in blood samples were extracted as somatic mutations. The list of identified mutations was further processed using in‐house scripts to remove low‐confidence and likely false positive mutations. Mutations fulfilling at least 1 of the following criteria were discarded as false positive: (1) quality score < 60, (2) depth of coverage < 20, (3) variant read observed in 1 strand only, (4) clipped sequence length < 100 (avg_clipped_length < 100), (5) variant located on either sequence end (avg_pos_as_fraction < 0.05), and (6) mutation matches 1 on an in‐house false‐positive list. Parameters specified in criteria (4) and (5) were calculated by bam‐readcount with option “‐q 1” (ver. 0.8.0) (https://github.com/genome/bam-readcount).

### 2.5 GEP using DNA microarray analysis

GEP analysis was performed in 892 (98.0%) samples, as previously described.^14^, ^17^ Total RNA was extracted from approximately 10‐mg tissue using an miRNeasy Mini Kit (Qiagen, Hilden, Germany) according to the manufacturer's instructions. RNA samples with an RNA integrity number of greater than or equal to 6 were used for DNA microarray analysis. Briefly, total RNA (100 ng) was amplified and fluorescently labeled. Labeled samples were hybridized to a SurePrint G3 Human Gene Expression 8 × 60 K v2 Microarray (Agilent Technologies, Santa Clara, CA). Data analysis was performed using GeneSpring GX software (Agilent Technologies). Raw signal intensity values were log transformed and normalized to the 75th percentile. The fold change between tumor and normal tissues from the same patient was calculated from the normalized values.

### 2.6 Tumor classification

In this study, CRCs were classified into 3 groups according to the single nucleotide variant (SNV) count and mutation spectrum, as previously reported from our institution and other institutions.^6^, ^14^ Briefly, CRCs exceeding 500 counts of nonsynonymous SNVs were classified as hypermutators, whereas the remaining CRCs were classified as nonhypermutators. Hypermutators were then subdivided into 2 groups according to nucleotide substitution frequency and pattern. CRCs that had more than 20% C‐to‐A and less than 3% C‐to‐G transversions were defined as POLE category tumors, whereas the remaining hypermutators were defined as common‐hypermutators.

### 2.7 Immunohistochemistry

The Immunohistochemistry (IHC) analysis for MMR proteins (MLH1, MSH6, MSH2, and PMS2) was performed to determine the tumor MMR status. In the current cohort, MMR status was analyzed only when MMR status was required for clinical practice and/or the patient provided consent for analyzing the MMR status**.** Accordingly, 76 of 910 (8.4%) tumors were investigated for MMR status. The resected specimens were fixed in 10% formalin, dehydrated, and embedded in paraffin. Paraffin sections (3‐µm thick) were used for IHC. The sections were pretreated with epitope retrieval solution 2 (Leica Biosystems, Newcastle, UK) for 40 min at 95°C and then reacted with antibodies specific for MLH1 (Clone ES05; Dako, Santa Clara, CA; dilution 1:50), MSH2 (Clone FE11; Dako; dilution 1:50), MSH6 (Clone EP49; Dako; dilution 1:50), and PMS2 (Clone EP51; Dako; dilution 1:25). After reaction with diaminobenzidine chromogen using EnVision + system‐ HRP Labelled Polymer Anti‐mouse (Dako), the slides were evaluated by pathologists. If the tumor showed the absence of tumor cells in at least 1 MMR protein, but retained expression in adjacent normal tissue as positive controls, the case was considered MMR‐deficient (MMR‐D).

### 2.8 Clinicopathological variables

Data on clinicopathological characteristics were collected from a prospective CRC database at Shizuoka Cancer Center Hospital. Right‐sided CRCs were defined as tumors arising from the cecum, ascending colon, or transverse colon. Left‐sided CRCs were defined as tumors arising from the descending colon, sigmoid colon, or rectum. After formalin fixation, tumor size was measured at its largest diameter. Disease pathological stage was defined in accordance with the International Union Against Cancer tumor lymph node metastasis classification.^18^

### 2.9 Statistical analysis

Statistical analyses were performed using BellCurve for Excel, version 2.15 (Social Survey Research Information Co., Ltd., Tokyo, Japan). Fisher's exact test was used to assess categorical variables. Mann‐Whitney U tests were used to compare continuous variables between 2 groups. Differences with *p* values of less than 0.05 were considered significant. This study is a retrospective exploratory study; thus, the multiplicity was not adjusted.

## 3 RESULTS

Patient and tumor characteristics are summarized in Table 1. In total, 910 primary CRC tissues were analyzed (Figure 1). The median age was 67 years (range: 20‐93 years). Small tumors tended to be excluded from Project HOPE since the removal of tumor tissue samples in patients with small tumors would make their pathological diagnosis difficult; therefore, most tumors (95.5%) were pT2 or more. The median tumor size was 45 mm (range: 14‐158 mm).

**Table 1:** Clinical characteristics of the patients

	N = 910
Sex	
Man	532 (58.5)
Woman	378 (41.5)
Age (years) [median (range)]	67 (20‐93)
Location	
Right	290 (31.9)
Left	620 (68.1)
Histology	
Well or moderately differentiated	854 (93.8)
Poorly differentiated or mucinous	56 (6.2)
Tumor size (mm) [median (range)]	45 (14‐158)
pT stage	
Tis	4 (0.4)
T1	37 (4.1)
T2	164 (18.0)
T3	398 (43.7)
T4	307 (33.7)
pStage	
0	4 (0.4)
I	158 (17.4)
II	276 (30.3)
III	357 (39.2)
IV	115 (12.6)

Note

Values represent numbers (percentages), unless indicated otherwise.

**Figure 2:**
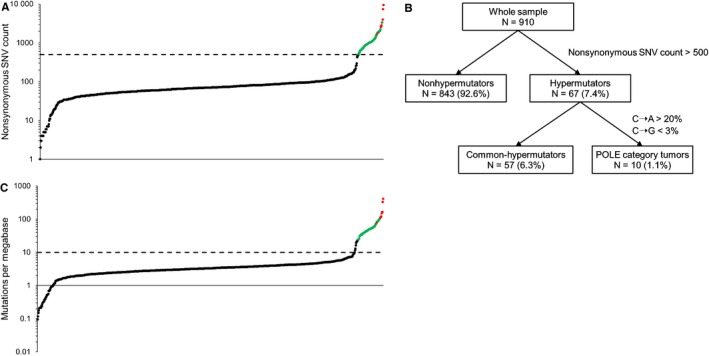
Sample classification. A, Distribution of nonsynonymous single nucleotide variant (SNV) counts. The broken line indicates a nonsynonymous SNV count of 500, and red and green circles indicate POLE category tumors and common‐hypermutators, respectively. B, All tumors were stratified into 3 groups according to the SNV count and mutation spectrum. C, Distribution of mutations/megabase. The broken line indicates a mutation rate of 10, and red and green circles indicate POLE category tumors and common‐hypermutators, respectively

Figure 2A shows the distribution of nonsynonymous SNV counts in all eligible samples. The median nonsynonymous SNV count was 71 (range: 1‐9515). Eight hundred forty‐three (92.6%) and 67 (7.4%) tumors were classified as nonhypermutators and hypermutators, respectively (Figure 2B). The hypermutators were then subdivided into 2 groups according to nucleotide substitution frequency and pattern. Among these hypermutators, 10 tumors (1.1% of all tumors) were classified as POLE category tumors. No hypermutators with more than 20% C‐to‐A transversions had greater than or equal to 3% C‐to‐G transversions. In addition, Figure 2C shows the distribution of mutations/Mb. All hypermutators had more than 10 mutations/Mb. Among nonhypermutators, 9 tumors had more than 10 mutations/Mb.

**Figure 3:**
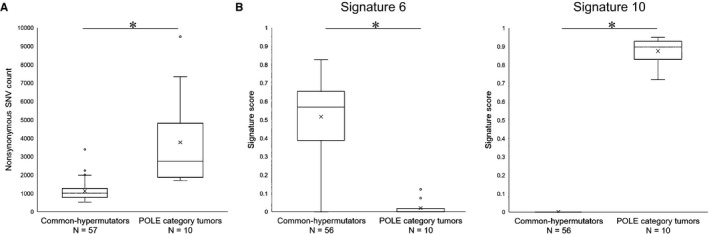
Comparisons between common‐hypermutators and POLE category. A, Comparison of nonsynonymous single nucleotide variant (SNV) counts. B, Comparison of signature scores for Signatures 6 and 10. *P < 0.05

Figure 3A shows a comparison of nonsynonymous SNV counts between common‐hypermutators and POLE category tumors. The SNV count was significantly higher in POLE category tumors than that in common‐hypermutators, suggesting that POLE category tumors had the ultramutated phenotype. Furthermore, using deconstructSigs,^19^ 30 mutational signatures of the COSMIC database were investigated in 56 of 57 common‐hypermutators and all POLE category tumors. Signatures 6 and 10 were related to microsatellite instability and *POLE* exonuclease domain mutation, respectively.^20^ As shown in Figure 3B, the signature score of Signature 6 was significantly higher in common‐hypermutators than in POLE category tumors, and the signature score of Signature 10 was significantly higher in POLE category tumors than in common‐hypermutators, suggesting the validity of the current tumor classification. No significant differences in signature score between common‐hypermutators and POLE category tumors were confirmed in other mutational signatures (data not shown).

Table 2 summarizes somatic mutations in the *POLE* gene leading to change in the amino acid sequence. In total, 27 (3.0%) tumors had somatic *POLE* mutations. All tumors belonging to the POLE category had exonuclease domain (86‐427; http://pfam.xfam.org/protein/Q07864) mutations in the *POLE* gene. Among POLE category tumors, 7 tumors contained P286R mutation, and we detected 1 F367C, 1 V411L, and 1 S297Y mutation in separate cases. In contrast, 4 other tumors having exonuclease domain mutations, such as K391T, Q125H, R259H, and Q196* mutations, did not belonged to the POLE category, and 2 tumors with K391T or Q196* were classified as common‐hypermutators. *POLE* mutations outside the exonuclease domain were confirmed in 13 tumors, and none were associated with POLE category tumors. Among these 13 tumors, 7 were classified as common‐hypermutators.

**Table 2:** Summary of somatic mutations in the *POLE* gene leading to changes in the primary structures of proteins

Case no.	*POLE* mutation	Mutation type	Exonuclease domain mutation	Nonsynonymous SNV count	Tumor classification
1	P286R	Missense	Yes	9515	POLE category
2	P286R	Missense	Yes	7351	POLE category
3	P286R	Missense	Yes	3963	POLE category
4	Y1813C	Missense	No	3385	Common‐hypermutator
5	P286R	Missense	Yes	3344	POLE category
6	P286R	Missense	Yes	2847	POLE category
7	F367C	Missense	Yes	2632	POLE category
8	P286R	Missense	Yes	2606	POLE category
9	P286R	Missense	Yes	1870	POLE category
10	V411L	Missense	Yes	1859	POLE category
11	P1207S, V1218I	Missense	No	1782	Common‐hypermutator
12	S297Y	Missense	Yes	1698	POLE category
13	E767D	Missense	No	1495	Common‐hypermutator
14	E1199D	Missense	No	1289	Common‐hypermutator
15	K879E, R1626H	Missense	No	1274	Common‐hypermutator
16	K391T	Missense	Yes	1120	Common‐hypermutator
17	T2049A	Missense	No	1110	Common‐hypermutator
18	Q196*	Nonsense	Yes	1041	Common‐hypermutator
19	R47W	Missense	No	723	Common‐hypermutator
20	R1289C	Missense	No	188	Nonhypermutator
21	T1904A	Missense	No	157	Nonhypermutator
22	V533M	Missense	No	119	Nonhypermutator
23	D1131E	Missense	No	97	Nonhypermutator
24	K1942*	Nonsense	No	60	Nonhypermutator
25	Q125H	Missense	Yes	51	Nonhypermutator
26	A1200T	Missense	No	45	Nonhypermutator
27	R259H	Missense	Yes	36	Nonhypermutator

Abbreviation: SNV, single nucleotide variant.

* Termination codon

Table 3 shows the tumor MMR status. MMR status was investigated in 76 of 910 tumors, including 49 of 843 (5.8%) nonhypermutators, 21 of 57 (36.8%) common‐hypermutators, and 6 of 10 (60.0%) POLE category tumors. All POLE category tumors were MMR proficient (MMR‐P), whereas all common‐hypermutators were MMR‐D. Two nonhypermutators were MMR‐D. One had 133 nonsynonymous SNVs and 6.0 mutations/Mb, and the other had 474 nonsynonymous SNVs and 21.0 mutations/Mb.

**Table 3:** Tumor mismatch repair status

Mismatch repair status	Nonhypermutators	Hypermutators	
		Common‐hypermutators	POLEcategory tumors
	N = 49	N = 21	N = 6
Mismatch repair proficient	47 (95.9)	0 (0)	6 (100)
Mismatch repair deficient	2 (4.1)	21 (0)	0 (0)

Note

Values represent numbers (percentages).

Table 4 shows a comparison of clinicopathological characteristics among nonhypermutators, common‐hypermutators, and POLE category tumors. Compared with patients with nonhypermutators and common‐hypermutators, those with POLE category tumors were significantly younger. Furthermore, POLE category tumors tended to be more common in men (vs nonhypermutators: *P* = 0.054; vs common‐hypermutators: *P* = 0.035). In addition, compared with nonhypermutators, POLE category tumors tended to be associated with poorly differentiated type, large tumor size, and early disease stage, although the differences were not statistically significant.

**Table 4:** Comparison of the clinicopathological characteristics of colorectal cancer by tumor classification

	Nonhypermutators (NH)	Hypermutators		P value NH vs PC	P value CH vs PC
		Common‐hypermutators (CH)	POLE category tumors (PC)		
	N = 843	N = 57	N = 10		
Age (years) [median (range)]	67 (20‐93)	69 (29‐87)	43 (30‐85)	0.002	0.007
Sex					
Man	494 (58.6)	29 (50.9)	9 (90.0)	0.054	0.035
Woman	349 (41.4)	28 (49.1)	1 (10.0)		
Location					
Right	245 (29.1)	41 (71.9)	4 (40.0)	0.489	0.069
Left	598 (70.9)	16 (28.1)	6 (60.0)		
Histology					
Well or moderately differentiated	805 (95.5)	41 (71.9)	8 (80.0)	0.076	0.717
Poorly differentiated or mucinous	38 (4.5)	16 (28.1)	2 (20.0)		
Tumor size (mm) [median (range)]	45 (14‐130)	55 (20‐152)	60 (30‐158)	0.029	0.379
pT stage					
Tis‐T2	190 (22.5)	13 (22.8)	2 (20.0)	1.000	1.000
T3‐T4	653 (77.5)	44 (77.2)	8 (80.0)		
pStage					
0‐II	392 (46.5)	38 (66.7)	8 (80.0)	0.052	0.487
III‐IV	451 (53.5)	19 (33.3)	2 (20.0)		
Lymphatic invasion (yes)	432 (51.2)	35 (61.4)	4 (40.0)	0.538	0.299
Vessel invasion (yes)	559 (66.3)	28 (49.1)	5 (50.0)	0.320	1.000

Note

Values represent numbers (percentages), unless indicated otherwise.

Patients with POLE category tumors were significantly younger than those with other CRCs, and the characteristics of patients with early‐onset POLE category CRCs (age of onset ≤50 years) were then investigated (Table 5). In total, 101 (11.1%) tumors were early‐onset CRCs in this study. Among early onset CRCs, 7 (6.9%) tumors were POLE category tumors. Notably, all mutations in *POLE* among early onset POLE category tumors were P286R.

**Table 5:** Characteristics of patients with early onset POLE category colorectal cancer (age of onset ≤ 50 years)

	N = 7
Age (years) [median (range)]	39 (30‐46)
Sex	
Man	6 (85.7)
Woman	1 (14.3)
Location	
Right	2 (28.6)
Left	5 (71.4)
Histology	
Well or moderately differentiated	5 (71.4)
Poorly differentiated or mucinous	2 (28.6)
Tumor size (mm) [median (range)]	60 (45‐158)
pT stage	
Tis‐T2	1 (14.3)
T3‐T4	6 (85.7)
pStage	
0‐II	5 (71.4)
III‐IV	2 (28.6)
Nonsynonymous SNV count [median (range)]	3344 (1870‐9515)
*POLE* mutation	
P286R	7 (100)

Note

Values represent numbers (percentages), unless indicated otherwise.

Abbreviation: SNV, single nucleotide variant.

**Figure 4:**
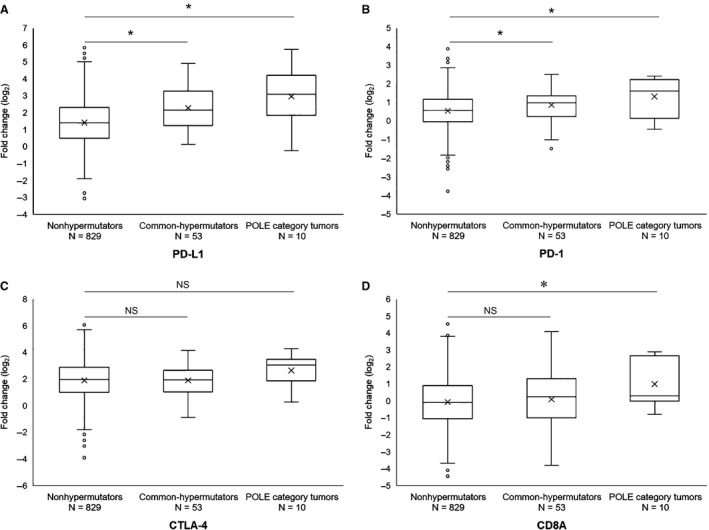
Association between the expression levels of genes associated with tumor immune responses and tumor classification. A, Comparison of *PD‐L1*. B, Comparison of *PD‐1*. C, Comparison of *CTLA‐4*. D, Comparison of *CD8A*. *P < 0.05

Previously, the association between high tumor mutation rate and response to immune checkpoint inhibitors was reported.^21^ Here, we examined the expression of genes associated with tumor immune response. GEP analysis was performed in 892 tumors, including 829 nonhypermutators, 53 common‐hypermutators, and 10 POLE category tumors. The expression of genes encoding immune checkpoint molecules, such as programmed cell death ligand 1 (PD‐L1), programmed cell death 1 (PD‐1), cytotoxic T‐lymphocyte antigen (CTLA)‐4, and CD8A, which is a marker of tumor‐infiltrating lymphocytes, was investigated. Compared with nonhypermutators, both common‐hypermutators and POLE category tumors exhibited significant upregulation of *PD‐L1* and *PD‐1* genes (Figure 4A, 4B). The 3 groups showed similar expression levels of *CTLA4* (Figure 4C). In contrast, *CD8A* was significantly upregulated in POLE category tumors compared with that in nonhypermutators (Figure 4D).

## 4 DISCUSSION

In this study, comprehensive WES was performed for 910 primary CRCs. We classified a small subset of CRCs (1.1% of all tumors) as POLE category tumors according to the nucleotide substitution frequency and pattern. POLE category tumors had mutations in the specific exonuclease domain of the *POLE* gene and harbored an extremely high TMB, suggesting that these mutations were associated with *POLE* proofreading deficiency.

In previous studies, tumors with pathogenic exonuclease domain mutations in the *POLE* gene were reported to harbor extremely high TMB, with a characteristic nucleotide base substitution exhibiting increased C‐to‐A transversions.^1^, ^7^ Moreover, a recent study showed that POLE category CRC and EC had carcinogenic mechanisms distinct from those of other tumors,^14^ suggesting that POLE category tumors may exhibit distinct characteristics. Therefore, we classified CRCs, according to SNV counts and the mutation spectrum, as previously described.^6^, ^14^ In the present cohort, 10 (1.1%) tumors were identified as POLE category tumors, all of which harbored mutations in the exonuclease domain at positions 286, 297, 367, or 411; these mutations have been reported to act as pathogenic mutation hotspots.^7^ No mutations outside the exonuclease domain were associated with POLE category tumors, and these mutations were considered passenger mutations caused by the accelerated mutational process. In addition, some exonuclease domain mutations in the *POLE* gene, such as Q125H, R259H, K391, and Q196*, were not associated with POLE category tumors. These mutations were also considered passenger mutations, whereas exonuclease domain mutations in *POLE* were previously reported to be harmful by in silico analysis.^22^ No novel pathogenic exonuclease domain mutations in *POLE* were identified in this study.

In addition, POLD1 has also been shown to have critical roles in proofreading by DNA polymerases.^3^ Previously, rare germline mutations in *POLE* and *POLD1* have been reported in patients with polymerase proofreading‐associated polyposis,^23^, ^24^ suggesting that germline *POLE* and *POLD1* mutations were involved in familial CRCs.^25^ However, it is still unclear whether somatic *POLD1* mutations act as drivers of spontaneous CRCs.^7^ In this study, although some tumors harbored the exonuclease domain of *POLD1* (data not shown), we did not examine whether these mutations were pathogenic.

When investigating the biological and clinical impact of *POLE* mutations, exonuclease domain mutations associated with POLE category tumors should be distinguished from other mutations because POLE category tumors harbor distinct carcinogenic mechanisms and mutation spectra and are therefore expected to be associated with distinct clinicopathological characteristics and clinical outcomes. Previously, several studies evaluated some exonuclease domain mutations in the *POLE* gene, although the associations between these mutations and proofreading deficiency were unclear.^22^, ^26^ This is of particular concern when comparing the biological and clinical characteristics of CRCs with exonuclease domain mutations in *POLE* across different studies. In addition, the selection criteria have varied among studies. For example, 1 study consisted of CRCs with only the microsatellite stable phenotype,^22^ whereas other studies consisted of predominantly stage II and III CRCs 27 or CRCs from young patients.^28^ Recently, several reports have demonstrated the clinicopathological characteristics of *POLE* mutant CRCs, and the frequency of exonuclease domain mutations was found to vary from 0.65% to 12.3%.^1^, ^22^, ^27^, ^28^, ^29^ This difference was due to the factors noted above. In the current study, we evaluated predominantly stage II and stage III CRCs, and CRCs treated with preoperative chemotherapy and/or radiotherapy were excluded. We demonstrated that patients with POLE category tumors were significantly younger than patients with nonhypermutators and common‐hypermutators. In addition, POLE category tumors tended to be more common in men. The patient selection in this study was relatively similar to that in a study by Domingo and colleagues.^27^ They focused on only pathogenic mutations in the *POLE* gene and divided CRCs into 3 groups, that is, MMR‐proficient (MMR‐P), MMR‐D, and *POLE*‐mutant groups. The frequency of the *POLE* mutant was 1.1%, and compared with the other 2 groups, the *POLE*‐mutant group was associated with younger age and male sex, similar to our current findings. These findings supported the reproducibility of these results in *POLE* mutant CRCs.

A link between high TMB and response to immune checkpoint inhibitors has been established.^21^ In CRCs, MMR‐D tumors, which have high TMB, are more responsive to PD1‐blockade than MMR‐P tumors, and a high TMB is associated with prolonged progression‐free survival.^30^ Previously, several reports have demonstrated dense immune infiltration in MMR‐D tumors,^31^, ^32^, ^33^ and MMR‐D tumors are predicted to have a large number of mutation‐associated neoantigens.^31^, ^34^, ^35^ Moreover, expression of multiple immunosuppressive molecules was highly upregulated in the MMR‐D CRC microenvironment.^33^ Collectively, these findings suggested that identifying antigenic neoepitopes generated by an exceptional number of tumor mutations is an important step triggering the efficient host antitumor immune response and that this immune response is counterbalanced by immune inhibitory signals. Therefore, immune checkpoint inhibitors are thought to be an attractive option in CRCs with pathogenic exonuclease domain mutations in the *POLE* gene, which harbors an extremely high TMB. In the current study, GEP analysis showed that PD‐L1 and PD‐1 were significantly upregulated in both common‐hypermutators and POLE category tumors compared with those in nonhypermutators. Additionally, *CD8A* was significantly upregulated in POLE category tumors compared with those in nonhypermutators. Somatic *POLE* mutations have also been reported to be associated with prominent T‐cell infiltrates in both precancerous and cancerous lesions and with an enhanced predicted clonal neoantigen burden.^36^ These findings suggested that POLE category tumors may be good candidates for immune checkpoint inhibitors.

Recently, Domingo and colleagues analyzed nearly 6,000 CRCs and reported similar prevalence and correlations of *POLE* mutations shown in this study. They also showed that patients with *POLE*‐mutant CRCs had reduced recurrence risk compared with those with other MMR‐P CRCs, suggesting that pathogenic mutations in the *POLE* gene may be prognostic biomarkers.^27^ In addition, POLE category tumors may be good candidates for immune checkpoint inhibitors, similar to MMR‐D tumors, as noted above. Therefore, it could be argued that the clinical significance of *POLE* proofreading deficiency in CRCs is relatively clear for personalized management. Currently, MMR status testing is relatively easy to perform. In western countries, universal screening is routinely performed to detect MMR‐D CRCs.^37^, ^38^ When the tumor is MMR‐P, the pathogenic exonuclease domain mutations in the *POLE* gene are worth investigating because tumors with *POLE* proofreading deficiency are MMR‐P.^1^, ^27^ Accordingly, searching for pathogenic mutations at recurrent hot spots may be clinically practical. Routinely applicable and straightforward molecular tests, such as polymerase chain reaction, may also be useful. In the future, antibodies for such exonuclease domain mutations may be developed for IHC. Furthermore, given that CRCs with pathogenic exonuclease domain mutations in *POLE* represent ~3% of all CRCs,^1^, ^25^, ^39^, ^40^ narrowing down candidates for analysis of *POLE* proofreading deficiency may be more useful for daily clinical practice. Our current findings suggested that these tests should be recommended for young patients. Similarly, Bourdais and colleagues suggested that *POLE* exonuclease domain mutation testing would be interesting in MMR‐P CRCs, particularly in young patients, for immune therapy, although they did not note which mutation would be the best candidate for the screening.^41^ In this study, the frequency of POLE category tumors was increased to 6.9% in early onset CRCs. Moreover, among early‐onset POLE category tumors, all *POLE* mutations were P286R. Therefore, searching for the P286R mutation in the *POLE* gene in young patients will be particularly valuable for daily clinical practice. In the future, additional studies in large populations are necessary to clarify the clinical significance of *POLE* proofreading deficiency analysis in the treatment of CRCs.

This study had several limitations. First, the number of CRCs belonging to POLE category tumors was relatively small, and the follow‐up periods after surgery were relatively short. Therefore, larger sample sizes and longer follow‐up durations are needed to further validate the current results and to investigate mid‐ or long‐term outcomes after surgery. Second, because most of the samples in this study were obtained from pT2 or more CRCs, the present results could not simply be extrapolated to smaller CRCs. However, most of the Tis or T1 CRCs were associated with a good prognosis and may not require specific therapies, such as immune checkpoint inhibitors. Third, the definition of the hypermutated type is still debatable, although we defined hypermutated tumors as previously described.^6^, ^14^ The definition of a hypermutated tumor in CRC has differed among studies, and further studies are necessary to establish a standard definition for this phenotype.

In summary, the subset of patients with CRC harboring pathogenic exonuclease domain mutations in the *POLE* gene had an extremely high TMB. These tumors had distinctive characteristics clinically and genetically. To the best of our knowledge, this is the largest study investigating the clinical significance of *POLE* mutations in Japanese patients with CRC, and the present findings can provide important insights into the development of personalized screening and management strategies for CRCs.

## CONFLICTS OF INTEREST

The authors declare that there are no conflicts of interest.
